# Clinical Protection of Goats against CpHV-1 Induced Genital Disease with a BoHV-4-Based Vector Expressing CpHV-1 gD

**DOI:** 10.1371/journal.pone.0052758

**Published:** 2013-01-03

**Authors:** Gaetano Donofrio, Valentina Franceschi, Angela Lovero, Antonio Capocefalo, Michele Camero, Michele Losurdo, Sandro Cavirani, Mariarosaria Marinaro, Erika Grandolfo, Canio Buonavoglia, Maria Tempesta

**Affiliations:** 1 Department of Medical-Veterinary Science, University of Parma, Parma, Italy; 2 Department of Veterinary Public Health, University of Bari, Valenzano, Italy; 3 Department of Infectious, Parasitic and Immune-Mediated Diseases, Istituto Superiore di Sanità, Roma, Italy; University of Southern California Keck School of Medicine, United States of America

## Abstract

Caprine herpesvirus type 1 (CpHV-1) is an alphaherpesvirus causing genital disease leading to abortion in adult pregnant goats and a systemic disease with high morbility and mortality in kids. Further, Caprine herpesvirus 1 infection represents a valuable large animal model for human herpesvirus induced genital disease, exploitable for pathogenic studies, new vaccines and antiviral molecules testing. Here, the bovine herpesvirus 4 (BoHV-4) based vector derived from an apathogenic isolate of BoHV-4 and expressing the immunodominant CpHV-1 glycoprotein D (BoHV-4-A-gD_cp_gD_106_ΔTK) was constructed and its ability to protect goats against CpHV-1 induced genital disease evaluated. The subcutaneous route of recombinant BoHV-4 administration was first tested *in vivo/ex vivo* by *in vivo* image analysis and *in vitro* by goat skin primary cultures preparation and transduction. Next, an exploratory immunization and safety study in goats was performed with two recombinant BoHV4, BoHV-4-A-gD_cp_gD_106_ΔTK or BoHV-4-CMV-IgK-gE2gD-TM. In both cases no clinical signs were evident but a good titer of serum neutralizing antibodies was produced in all inoculated animals. When a challenge experiment was performed in a new group of animals using a highly pathogenic dose of CpHV-1, all the vaccinated goats with BoHV-4-A-gD_cp_gD_106_ΔTK were protected toward CpHV-1 induced genital disease respect to the unvaccinated control which showed typical vaginal lesions with a high grade of clinical score as well as a long lasting viral shedding. In summary, the data acquired in the present study validate BoHV-4-based vector as a safe and effective viral vector for goat vaccination against CpHV-1 induced genital disease and pave the way for further applications.

## Introduction

Caprine herpesvirus 1 (CpHV-1) is a virus belonging to the *Herpesvirales* order, *Herpesviridae* family, *Alphaherpesvirinae* sub-family and *Simplexvirus* genus [1]. CpHV-1 is correlated with two different clinical entities in goats: a lethal systemic disease in kids [2] and a genital disease leading to balanoposthitis [3], vulvovaginitis [4] and abortion [5] in adults. Although CpHV-1 full genome sequence is not available yet, CpHV-1 restriction site maps were constructed by double digestion and cross hybridization of single restriction fragments. CpHV-1 mol. wt. DNA, as calculated by summation of the mol. wt. of single fragments, deriving from digestions with various endonucleases is ∼137 kbp [6].

From the pathogenic point of view, CpHV-1 infection starts locally at the respiratory or genital tract and successively, through a mononuclear cell-associated viremia, the virus spread systemically causing abortion in pregnant animals. The virus can be excreted via ocular, nasal and genital route. The genital apparatus is considered to be the most important site for virus entry and maintenance of infection in the herd [7]. In kids, CpHV-1 causes a systemic disease characterized by high morbidity and mortality rates, where ulcerative and necrotic lesions are distributed throughout the enteric tract. In adult goats, the infection leads to vulvovaginitis or balanoposthitis. Abortions associated with CpHV-1 occur during the second half of pregnancy and can be experimentally reproduced after intranasal or intravenous inoculation of pregnant goats [8]–[10]. Sacral ganglia are the primary CpHV-1 latent site following intra-vaginal infection. CpHV-1 reactivation can occur by physiological stress during the mating season and the hormonal status at oestrus could play a role. Whereas, experimental reactivation is difficult and requires the use of high doses of dexamethasone [11]. Interestingly, CpHV-1 shares several biological features with HSV-2 and BoHV-1, such as, the molecular features, the tropism for the vaginal epithelium, the type of genital lesions and the establishment of latency in sacral ganglia [6], [12].

In contrast to CpHV-1, bovine herpesvirus 4 (BoHV-4) is a gammaherpesvirus. BoHV-4 has been isolated from a variety of samples and cells from healthy cattle and from cattle that have experienced abortion or with metritis, pneumonia, diarrhoea, respiratory infection, and mammary pustular dermatitis [13]. Although the idea that BoHV-4 is involved in bovine post-partum metritis, albeit only as a secondary agent along with other agents like bacteria, is becoming consistent [14], [15], however the pathogenic role of BoHV-4 remains controversial. BoHV-4 has been classified as a gammaherpesvirus based on its genome sequence [16], however it differs from other gammaherpesviridae members in important biological properties. Unlike most other gammaherpesviruses, BoHV-4 causes cytopathic effect (CPE) and replicates in a variety of primary cultures and cell lines of bovine and various other animal species. In addition, there is no evidence for oncogenicity or growth transformation by BoHV-4. BoHV-4 has the ability to accommodate large amounts of foreign genetic material within its genome without any appreciable detrimental effect on its replication. For these reasons, it has been proposed as a viral vector for gene delivery and cancer therapy [17]–[23].

CpHV-1 infection is distributed worldwide and major economic losses, often underestimated, occur in Mediterranean countries and no vaccines are commercially available. The use of recombinant viral vaccines, although still far from reality, seems to be very promising in terms of their safety and efficacy, and BoHV-4 based vector, due to its biological characteristics, has good chance to be one the best candidate.

In the present paper, starting from a non-pathogenic strain of BoHV-4 isolated from the cell milk fraction of a healthy cow and its genome cloned as a bacterial artificial chromosome (BAC) [20], a recombinant BoHV-4 delivering the CpHV-1 glycoprotein D (gD) was constructed and proven to be immunogenic and protective against CpHV-1-induced pathology in goats.

## Results

### Design and expression of CpHV-1 gD tagged peptide

**Figure 1:**
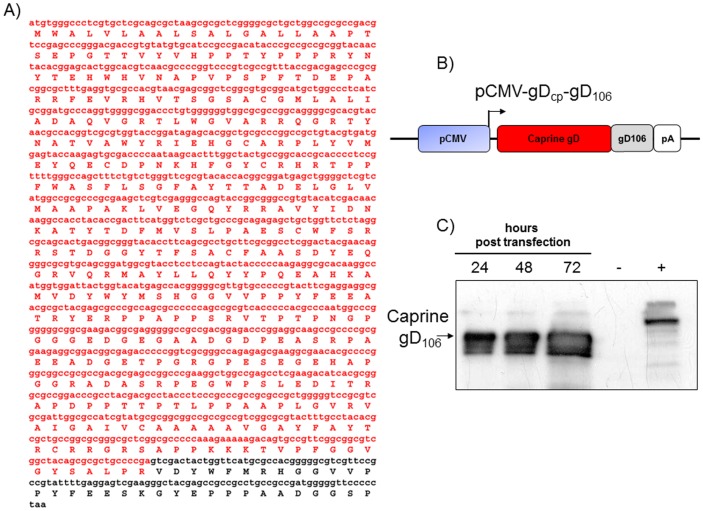
**A**) gD_cp_gD_106_ peptide sequence and predicted amino-acid product. In red CapHV-1 gD whereas in black the gD_106_ tag. **B**) Diagram (not to scale) showing the pCMV-gD_cp_gD_106_ construct: In blue the cytomegalovirus enhancer promoter (CMV), in red the CapHV-1 gD, in black the gD_106_ tag and in white the bovine growth hormone polyadenylation signal (PA). **C**) western immune-blotting of pCMV-gD_cp_gD_106_ transfected HEK cells lysates at different time post transfection (24, 48 and 72 hours). Untransfected HEK cells as negative control (−) and pIgKE2gD-TM transfected HEK cells lysate as a positive control (+).

Being CpHV-1 gD an immunodominant antigen and responsible for mounting a serum neutralizing humoral immune response in naturally and experimentally infected goats [24], [25], it was chosen as an ideally antigen to be expressed by BoHV-4 based vector. The full length CpHV-1 gD ORF was amplified by PCR from CpHV-1 infected cells DNA and sub cloned in-frame with a tag peptide, gD_106_ [26] (**Fig. 1A**), in a eukaryotic expression vector and pCMV-gD_cp_gD_106_ was generated (**Fig. 1B**). pCMV-gD_cp_gD_106_ contains the CMV promoter, the CpHV-1 gD ORF fused with gD_106_ and the bovine growth hormone polyadenylation signal (**Fig. 1B**). pCMV-pCMV-gD_cp_gD_106_ functionality was assessed in transiently transfected HEK 293T cells, by Western immune-blotting. As shown in **Fig. 1C**, gD_cp_gD_106_ was abundantly expressed.

### Generation of a BoHV-4 based vector expressing pCMV-gD_cp_gD_106_

gD_cp_gD_106_ was excised out from p-CMV-gD_cp_gD_106_ and sub-cloned in pINT2 [17], a plasmid vector containing two BoHV-4 TK gene sequences, to obtain pTK-CMV-gD_cp_gD_106_-TK. Next, TK-CMV-gD_cp_gD_106_-TK was cut out from the plasmid backboone and electroporated in SW102 *E. coli* containing the pBAC-BoHV-4-A-KanaGalKΔTK. pBAC-BoHV-4-A-KanaGalKΔTK is a BoHV-4 genome clone coming from a non-pathogenic strain of BoHV-4 isolated from the milk cell fraction of an healthy cow, who genome was cloned as a Bacterial artificial Chromosome (BAC), pBAC-BoHV-4-A; and where its TK locus was targeted with a KanaGalK selectable cassette [20].

**Figure 2:**
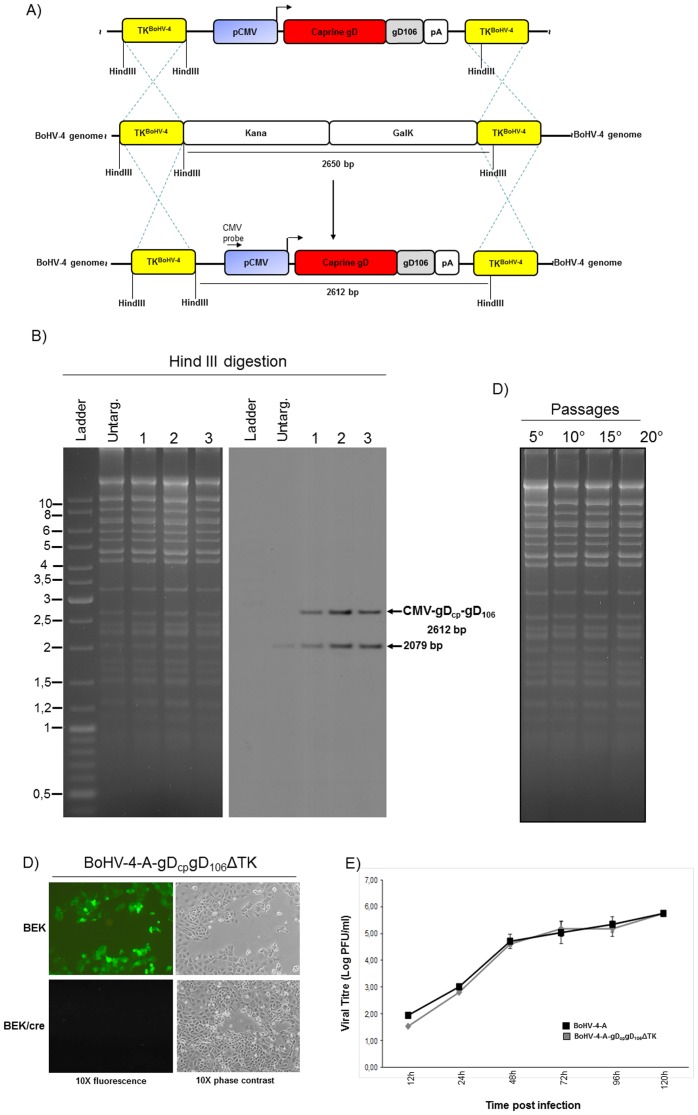
**A**) Diagram showing (not to scale) the targeting by heat-inducible homologous recombination in SW102 containing pBAC-BoHV-4-A-TK-KanaGalK-TK, where the Kana/GalK cassette was removed and replaced with the CMV-gD_cp_gD_106_ expression cassette. **B**) Representative colonies tested by HindIII restriction enzyme analysis, agar gel electrophoresis, and Southern blotting performed with a probe for the CMV promoter sequence, where the 2,612 bp band is present only for the targeted clones bat not for the untargeted control. **C**) Stability of the pBAC-BoHV-4-A-gD_cp_gD_106_ΔTK plasmid in *E. coli* SW102 cells. **D**) Representative fluorescent microscopic images of plaques formed by viable reconstituted recombinant BoHV-4-A-gD_cp_gD_106_ΔTK after DNA electroporation into BEK cells or in BEK cells expressing cre recombinase. Magnification, ×10. **E**) Replication kinetics of BoHV-4-A-gD_cp_gD_106_ΔTK growth on cre-expressing cells, compared with those of the parental BoHV-4-A isolate. The data presented are the means ± standard errors of triplicate measurements (*P*>0.05 for all time points as measured by Student's *t* test).

TK-CMV-gD_cp_gD_106_-TK electroporated *E. coli* containing the pBAC-BoHV-4-A-KanaGalKΔTK was first heat-induced, negative selected on deoxygalactose minimal plates and then the resulting clones were negative selected with medium containing kanamicine. Therefore, retargeting was performed to the same site to replace the KanaGalK cassette with the CMV-gD_cp_gD_106_ cassette to obtain pBAC-BoHV-4-A-gD_cp_gD_106_ΔTK (**Fig. 2A**). Retargeted clones were analyzed by HindIII restriction enzyme and southern Blotting with a specific probe for CMV promoter. Although two hybridization bands appeared on the autoradiogram, it was due to the presence of CMV promoter either into the BAC backboone bringing an EGFP expression cassette or into CMV-gD_cp_gD_106_. However, the targeted clones were well distinguishable from the untargeted control because the later one displayed a single band (**Fig. 2B**). The selected clones' stability was assessed by serially passaging over 25 days and analysis by HindIII restriction enzyme digestion (**Fig. 2C**).

Infectious virus, BoHV-4-A-gD_cp_gD_106_ΔTK, was then reconstituted electroporating pBAC-BoHV-4-A-gD_cp_gD_106_ΔTK into BEK*cre* cells expressing the cre recombinase [20] to eliminate the BAC cassette from the viral genome (**Fig. 2D**). When BoHV-4-A-gD_cp_gD_106_ΔTK and its parental virus BoHV-4-A were compared in terms of replication kinetic, no appreciable differences were observed between them (**Fig. 2E**). Therefore, the introduction of the CMV-gD_cp_gD_106_ heterologous expression cassette into BoHV-4 genome TK locus does not confer detrimental effects as previously shown for other heterologous expression cassettes [17], [20], [21], [27].

### In vivo and ex vivo assessment of BoHV-4 transduction in goats by in vivo image analysis

**Figure 3:**
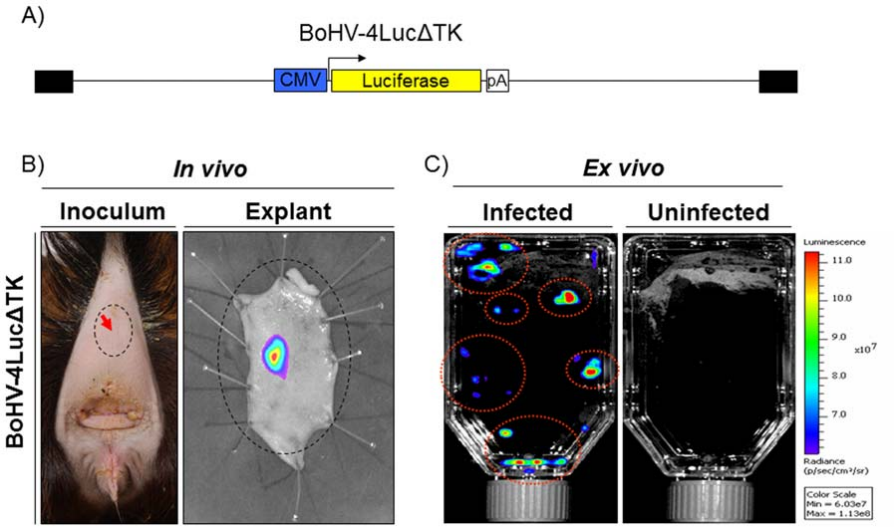
**A**) Diagram not on scale of BoHV-4LucΔTK delivering the CMV-Luc expression cassette. CMV promoter is represented by the blue box, the luciferase ORF by the yellow box whereas the polyadenylation signal by the white box. **B**) Representative image of the site of BoHV-4LucΔTK injection, under the goat tail (red arrow), and the skin explant visualized by *in vivo* image analysis. **C**) Representative image of flasks containing the BoHV-4LucΔTK infected or uninfected goat skin explant organotypic cultures and visualized by *in vivo* image analysis.

Before to attempt the use of BoHV-4 based vector for vaccination purposes in goats, it was of interest to investigate the BoHV-4 based vector competence to transduce goat tissue *in vivo* and *ex vivo*. A recombinant BoHV-4 expressing the luciferase enzyme under the control of the CMV promoter, BoHV-4-A-LucΔTK (**Fig. 3A**), was constructed replacing the KanaGalK selectable cassette in pBAC-BoHV-4-A-KanaGalKΔTK with CMV-Luc expression cassette (paper in preparation). BoHV-4-A-LucΔTK was subcutaneously injected under the tail of a goat and the skin comprising the site of injection and the surrounding area was surgically explanted, treated with a luminescent substrate and monitored by *in vivo* image analysis 48 hours post injection. As observable in **Fig. 3B**, a strong fluorescent signal was localized to the site of BoHV-4-A-LucΔTK injection, demonstrating that BoHV-4LucΔTK transduced the cells of the injected site and the luciferase transgene was expressed. An identical result, corroborating the above data, was obtained *ex vivo* (**Fig. 3C**) when organotypic cultures were first prepared from goat skin explant and subsequently infected with BoHV-4-A-LucΔTK.

### Assessment of BoHV-4 transduction in goat primary cell cultures

**Figure 4:**
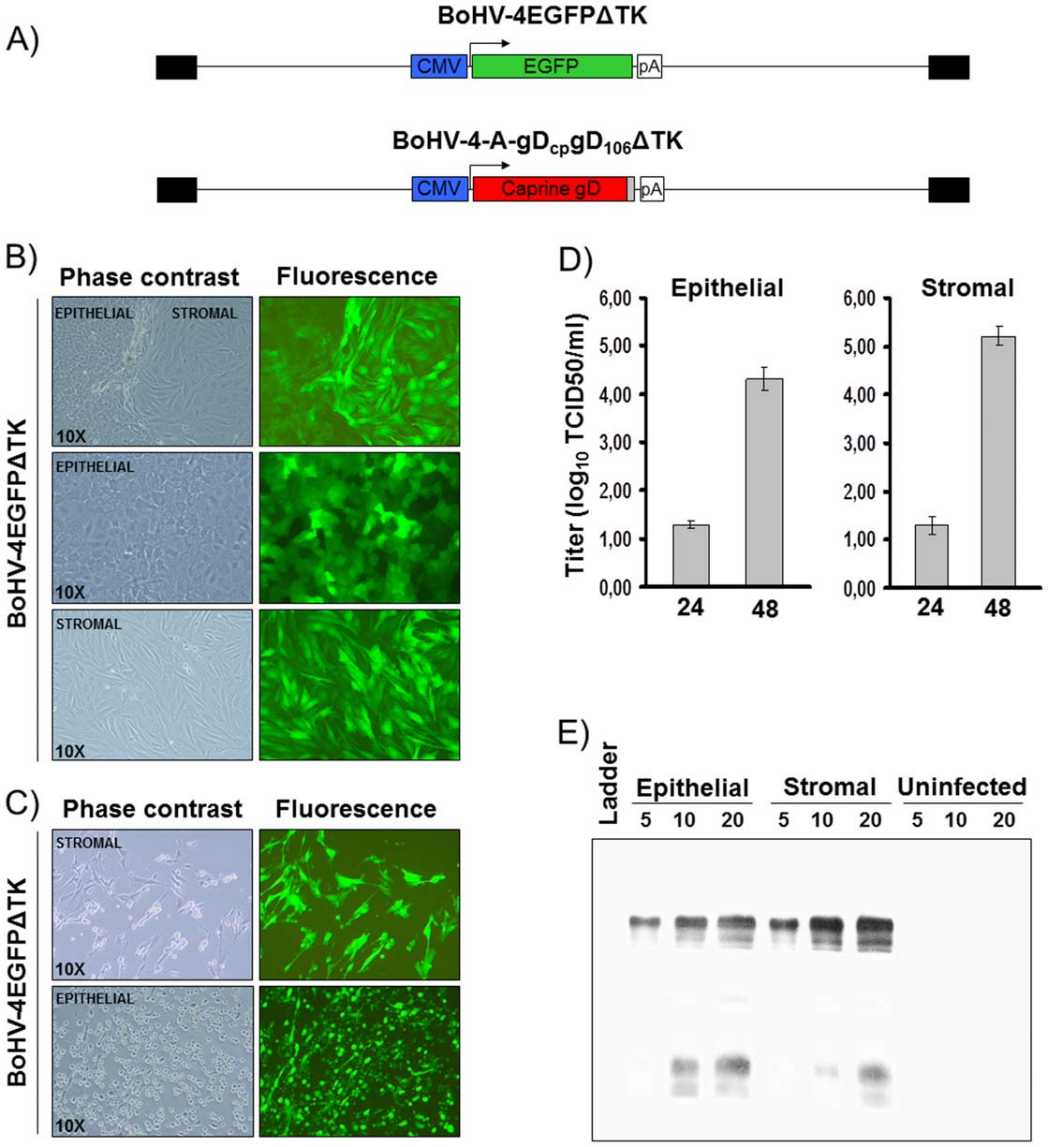
**A**) Diagram not on scale of BoHV-4EGFPΔTK delivering the CMV-EGFP expression cassette and BoHV-4-A-gD_cp_gD_106_ΔTK delivering the CMV-gD_cp_gD_106_ expression cassette. CMV promoter is represented by the blue box, the enhanced green fluorescent protein ORF by the green box, the caprine gD ORF by the red box, the gD_106_ tag by the grey box, whereas the polyadenylation signal by the white box. **B**) Representative phase contrast and fluorescence images of BoHV-4EGFPΔTK infected goat subcutaneous primary cell cultures at 24 hours post infection. **C**) Representative phase contrast and fluorescence images of BoHV-4EGFPΔTK infected goat subcutaneous primary cell cultures at 48 hours post infection and showing cytopathic effects. **D**) BoHV-4EGFPΔTK titer at 24 and 48 hours post infection as log_10_ TCID50/ml. The data presented are the means ± standard errors of triplicate measurements (*P*>0.05 for all time points as measured by Student's *t* test). **E**) Western immunoblotting of BoHV-4-A-gD_cp_gD_106_ΔTK infected goat subcutaneous epithelial and stromal primary cell cultures lysates. 5, 10 and 20 are the different amount as µl of the lysates.

Starting from the encouraging results obtained by *in vivo* image analysis, to better characterize goat subcutaneous cell transducible by BoHV-4 based vector, primary cell cultures from goat skin explant were prepared. At the beginning of the primary cell cultures preparation, mixed populations of epithelial and stromal cell type were obtained; however after few passages homogeneous cultures of epithelial and stromal cells were obtained. Mixed as well as pure population of goat subcutaneous epithelial end stromal primary cell culture were infected with a recombinant BoHV-4 expressing green fluorescent protein, BoHV-4EGFPΔTK [17] (**Fig. 4A**), to rapid monitor the cell transduction by GFP expression under fluorescence microscopy. As fast as 24 hours post infection both epithelial and stromal cells were nicely transduced by BoHV-4EGFPΔTK as shown by GFP expression (**Fig. 4B**) and after 48 hours post infection the CPE interested the cell monolayers in both epithelial and stromal cells (**Fig. 4C**); therefore indicating the replication competence of BoHV-4 in those cells. In fact when the virus was tittered a shift of the virus titer was observed between 24 and 48 hours post infection (**Fig. 4D**). Moreover, when the cells were infected with BoHV-4-A-gD_cp_gD_106_ΔTK (**Fig. 4A**) and analyzed by western immunoblotting they expressed gD_cp_gD_106_ (**Fig. 4E**).

### BoHV-4-A-gDcpgD106ΔTK an BoHV-4-CMV-IgK-gE2gD-TM inoculated goats produces specific serum neutralizing antibodies against the homologous virus

**Figure 5:**
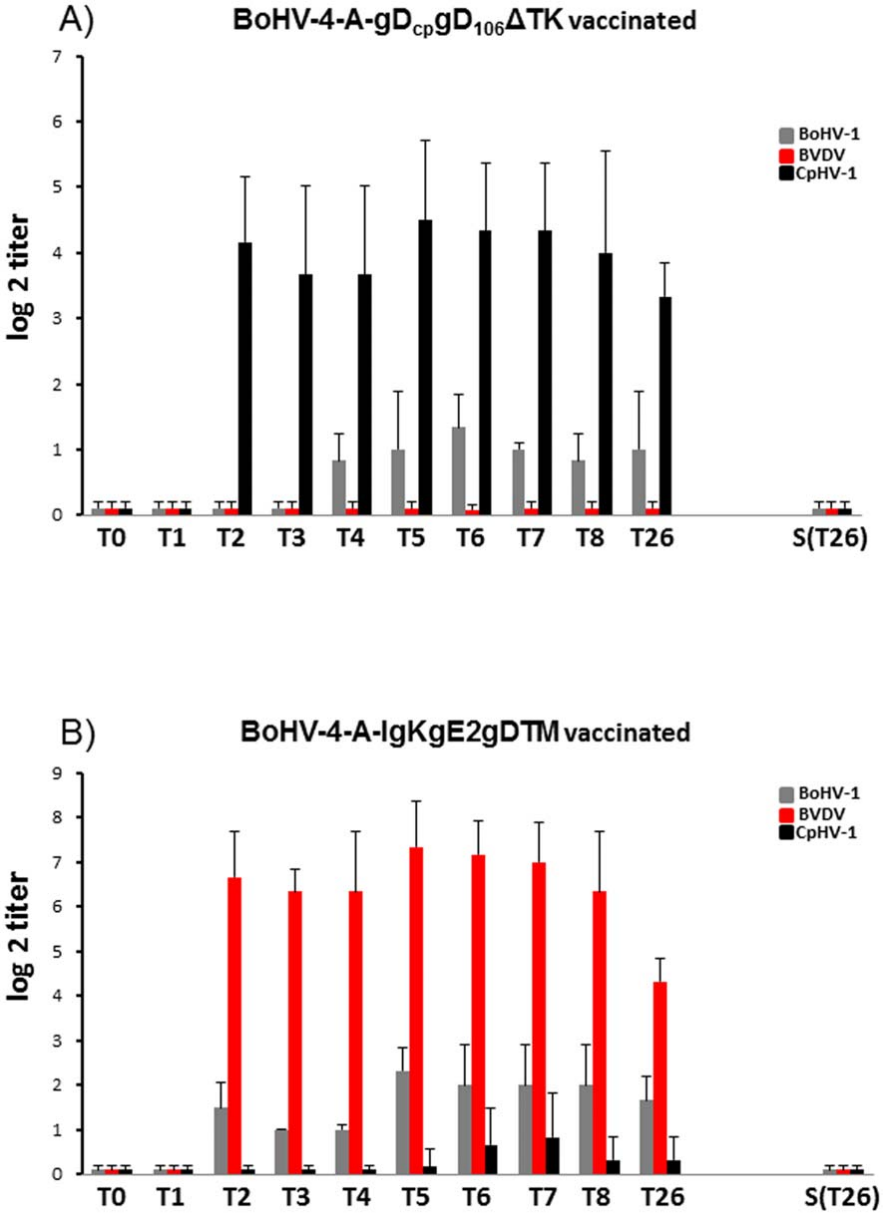
**A**) Kinetics of the humoral immune responses of goats immunized with BoHV-4-A-gD_cp_gD_106_ΔTK and BoHV-4-A-CMV-IgK-gE2gD-TM (**B**). Anti-BoHV-1 [Bovine herpesvirus 1 (grey bars)], anti-BVDV [Bovine viral diarrhea virus (red bars)] and anti-CapHV-1 [Caprine herpesvirus 1 (black bars)] serum-neutralizing antibodies (SN) as measured by serum neutralization test. SN antibodies were expressed as the reciprocal of the highest dilution of the serum that inhibited the development of virus-induced CPE in MDBK cells. Virus neutralization titers of >2 (log_2_) were considered positive. Each value represents the mean response of five rabbits ± the standard error of the mean (*, *P*<0.005 as measured by Student's *t* test or one-way analysis of variance). T1, T2, T3, T4, T5, T6, T7, T8 and T26 is the time (T) expressed as week (1,2,3,4,5,6,7,8 and 26) post inoculation. S(T26) is the sentinel animal at 26 weeks. T0 is the pre immune sera.

For exploring the ability of recombinant BoHV-4 to induce a neutralizing antibody immune response, a pilot immunization of goats was performed. In accordance with Italian laws on animal experimentation, which suggest minimizing the number of animals used, 7 BVDV, BoHV-1, and CpHV-1-serum-negative goats were used for subcutaneous inoculation of BoHV-4-A-gD_cp_gD_106_ΔTK or BoHV-4-CMV-IgK-gE2gD-TM, a recombinant BoHV-4 expressing a gE2gD chimeric peptide, made by fusion the ectodomain of the BVDV glycoprotein E2 (gE2) with the full length of the BoHV-1 glycoprotein D (gD) [21]. After collection of pre-immune serum, 3 goats were subcutaneously inoculated with 1 ml of 10^6^ TCID_50_/ml of BoHV-4-CMV-IgK-gE2gD-TM, 3 with 10^6^ TCID_50_/ml of BoHV-4-A-gD_cp_gD_106_ΔTK and 1 goat was not inoculated and left into the group as a sentinel animal to verify if the recombinant virus could spread through the animals. An identical inoculation was performed 2 weeks later. Blood samples were collected weekly from all animals for the assessment of anti-BoHV-1, anti-BVDV and anti-CapHV-1 antibodies by serum neutralization test. All the inoculated animals developed an antibody response, detectable in the second week after the first viral inoculation. Because the antibody titer was observable 2 weeks following the first viral inoculation and the blood samples were collected before the second viral inoculation, it was possible to note that a single inoculation of BoHV-4-A-gD_cp_gD_106_ΔTK or BoHV-4-CMV-IgK-gE2gD-TM was able to elicit a serum neutralizing humoral immune response (**Fig. 5**). However, goats inoculated with BoHV-4-A-gD_cp_gD_106_ΔTK developed serum neutralizing antibodies mainly against CpHV-1, a low amount of cross neutralizing antibodies against BoHV-1 and no serum neutralizing antibody against BVDV (**Fig. 5A**). Whereas, goats inoculated with BoHV-4-CMV-IgK-gE2gD-TM developed serum neutralizing antibodies mainly against BVDV, BoHV-1 and a low amount of cross neutralizing antibodies against CpHV-1 (**Fig. 5B**). The sentinel animal did not developed serum neutralizing antibodies against all viruses tested (**Fig. 5A and B**) and none of the animals displayed body temperature elevation or other clinical signs of disease during the time observation window of this study (data not shown).

As revealed by immunofluorescence assays (not shown), while anti-BoHV-4 antibodies were produced by all animals, no neutralizing antibodies directed against BoHV-4 could be detected in serum samples from both groups of animals.

### BoHV-4-A-gDcpgD106ΔTK inoculated goats are clinically protected against CpHV-1 challenge

To evaluate the protective efficacy of BoHV-4-A-gD_cp_gD_106_ΔTK toward CpHV-1 challenge, a challenge experiment was performed.

Three goats (A, B, C) were randomly chosen and subcutaneously vaccinated twice 15 days a part with BoHV-4-A-gD_cp_gD_106_ΔTK. One goat (D) was kept unvaccinated as negative control. Ten days after the second vaccination, all the goats were challenged intravaginally with 4 ml of virulent BA.1 strain of CpHV-1 (titer 10^5^TCID_50_/50 µl).

**Figure 6:**
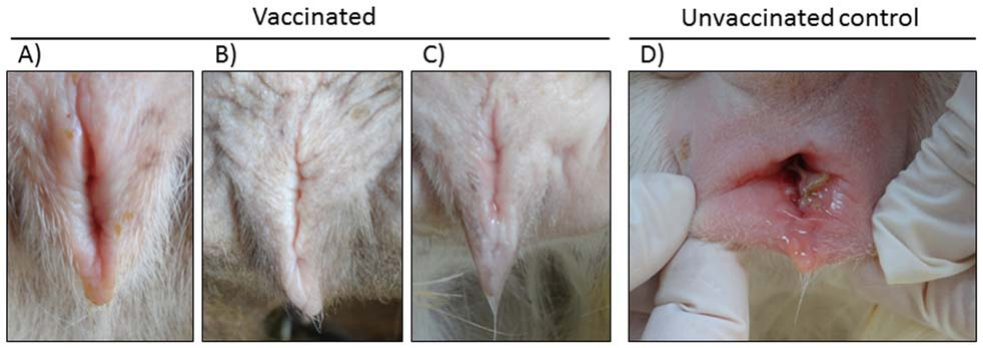
Representative image of typical CpHV-1 induced vaginal lesions in unvaccinated goat (D) respect to vaccinated goats (A, B, C).

**Figure 7:**
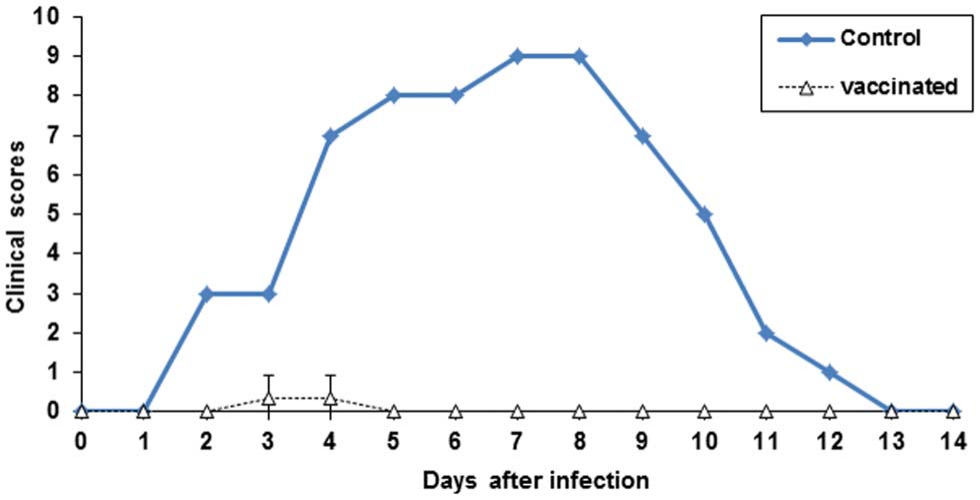
Clinical scores of vaccinated and control goats following CpHV-1 vaginal challenge.

During and after the vaccinations the goats did not have any general or local clinical signs. Following the challenge, only one of the vaccinated goats (**Fig. 6A**) had a light vaginal hyperemia for 2 days (score 1). There were no oedema and vesicular-ulcerative lesions or local pain in any of the vaccinated goats (**Fig. 6 A, B and C**). Control goat, as previously observed [28], showed typical CpHV-1 signs such as fever lasting six days and reaching peaks of 40.5°C, severe vaginal hyperaemia lasting 11 days, vulvar oedema and ulcers healing in 12 days, fibrinous vaginal discharge with blood spots for 5 days accompanied by pain at swabbing (**Fig. 6D**). The total clinical scores of the three vaccinated goats (A, B, C) were 2, 0 and 0 respectively. As reported in **figure 7**, the median AUC value of clinical scores of the vaccinated goats was 0,666 whereas that of the control goat was 62.

### Viral shedding

**Figure 8:**
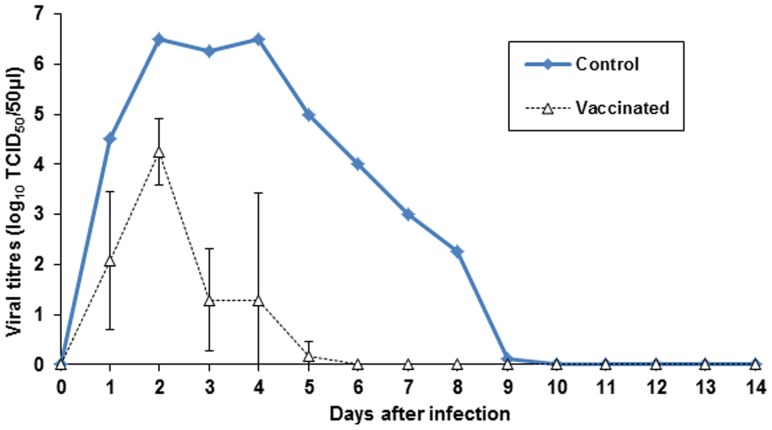
Virus titers in vaginal swabs of vaccinated and control goats after CpHV-1 challenge.

As reported in **figure 8**, vaccinated-challenged goats shed virus from vagina from the first to the fifth day post challenge (dpc). In particular one goat (A) shed the virus for five days; another (B) for three and another (C) for four days with a peak of viral shedding at dpc 2 (10^5^, 10^4^, 10^3,75^ TCID_50_/50 µl respectively). The control goat (D) shed the virus from the first to the ninth dpc, with a peak of viral excretion at day 2 and 4 dpc (10^6.50^ TCID_50_/50 µl). The AUC values were 13.75, 6.5 and 6.99 for the vaccinated goats and 38.12 for the control goat. The median AUC values were 9,08 for the vaccinated goats and 38,12 for the control goat indicating significant difference (P-value** = 0.01537** *P*<0.05) .

Viral DNA copies were detected by Real-time PCR in the vaginal swabs up to 7–8 dpc in vaccinated goats and to 12 dpc in the control goat (data not shown).

In vaccinated goats, SN antibodies were first detected fifteen days after the first vaccination and titers ranged from 1∶2 to 1∶8. They arise to 1∶8, 1∶16 at the time of the challenge (10 days after the second vaccination) and the titers reached the peak at day 32 post vaccination (7 days after challenge). The control goat, seroconverted 7 days after the challenge with a titer of 1∶16.

## Discussion

BoHV-1 and CpHV-1 genome have the typical arrangement of herpesvirus class D genome, an high degree of sequence homology as well as a serological cross reactivity [6]. Due to these similarities and considering that CpHV-1 is able to experimentally cross the species barrier and establish infection in calves [29], a live attenuated glycoprotein E (gE) negative BoHV-1 was proposed to be a candidate vaccine against CpHV-1 genital infection and shown to confer a partial protection [30].

BoHV-1 gD is involved in the virus entry process, which is conventionally divided into two steps: the initial attachment via interaction of viral attachment proteins with receptors on the cell surface and subsequent penetration involving membrane fusion. Antibodies raised against gD can lead, thereby, to a serum-neutralizing effect on the virus, and the presence of neutralizing antibodies in vaccinated animals are considered a better predictive indication for vaccine efficacy respect to the cell mediate immunity. In fact BoHV-1 gD has been extensively used for vaccine development and its ORF has been genetically manipulated and expressed by prokaryotic and eukaryotic systems with variable results [21], [31], [32]. CpHV-1 gD ORF is 1224 nucleotides long and encodes for a protein of 407 amino acids with a predicted molecular mass of 44.3 kDa, a signal peptide comprising the first 17 amino acids, an hydrophobic region (amino acids 360–376) corresponding to its transmembrane domain near the carboxy terminus and two potential *N*-linked glycosylation sites (positions 41 and 102) [24]. However beyond CpHV-1 gD molecular characteristics no attempt concerning the use of CpHV-1 gD as an immunogen against CpHV-1 induced genital infection has been performed so far.

Based on these information, in this paper, CpHV-1 gD ORF was first cloned, tagged at its carboxy terminus and then expressed as a membrane-linked (ML) form. The ML form was preferred respect to the secreted form, where CpHV-1 gD transmembrane domain is removed, because it was previously shown that heterologous glycoproteins expressed as ML form, by BoHV-4-based vector infected cells, induced a higher level of serum neutralizing antibody response [21], [33]. The reason for such phenomena, in terms of effective immune response, could resides in the presence of the transmembrane domain and raising two hypotheses: i), the gD transmembrane domain could display specific determinant epitopes for the formation of serum-neutralizing antibodies; ii), the presence of the transmembrane domain in the carboxy terminus of the gD, targeting the protein to the cellular surface, could allow a better recognition and processing of the antigen from the antigen presenting cells of the immune system.

A recombinant BoHV-4, BoHV-4-A-gD_cp_gD_106_ΔTK, delivering a CMV-gD_cp_gD_106_ expression cassette into the TK locus, was successfully obtained by recombination-mediated genetic engineering. The reconstituted virus in eukaryotic cells abundantly expressed gD_cp_gD_106_ chimeric peptide, which was shown to be stable, at least *in vitro* in terms of transgene expression, through the passages. Although BoHV-4 is considered a virus without a clear disease association, the existence of a BoHV-4 biotype potentially pathogenic cannot be absolutely excluded when a virus is going to be exploited as a gene delivery vector. For this reason a non-pathogenic strain of BoHV-4 isolated from the cell milk fraction of a healthy cow and its genome cloned as a bacterial artificial chromosome (BAC) [20] was used.

Appropriate vaccine administration is a critical component of a successful immunization program. The recommended route and site of inoculation for each vaccine are based on clinical trials, practical experience and theoretical considerations considering that a wrong route of administration may reduce the efficacy of the vaccine or increase local adverse reactions mainly when a replicating competent viral vector is going to be employed as in the present study. Because no data concerning goats immunization with BoHV-4-based vector are available, it was of interest to investigate the fate and the transduction capability of BoHV-4 based vector through the sub-cutaneous route of inoculation. Tacking advantage by a BoHV-4 delivering a luciferase expression cassette, it was possible to show BoHV-4 ability to transduce subcutaneous cells by in-vivo bioluminescent imaging (BLI). BLI is a very versatile and sensitive tool that is based on detection of light emission from cells or tissues and is specific for the analysis of cellular and biological processes [34]. In deed it was possible to visualize in vivo and ex vivo BoHV-4 gene delivery at the site of inoculation. Further, using skin cells organotypic cultures, a BoHV-4 expressing GFP and BoHV-4-A-gD_cp_gD_106_ΔTK it was deduced that both skin epithelial and stromal cells were susceptible to BoHV-4 infection/transduction. These data, although simple, are very informative and meaning that the sub-cutaneous route of BoHV-4-based vector inoculation is prone to antigen production.

As a preliminary immunization and safety study, before to attempt a challenge experiment, two groups of goats were subcutaneously inoculated with BoHV-4-A-gD_cp_gD_106_ΔTK or BoHV-4-CMV-IgK-gE2gD-TM respectively and all animals got successfully immunized as expected. In agreement with a previous study [35], the homologous antigen gave a better long lasting response in terms of neutralizing antibody titers. But the most interesting observation, concerning BoHV-4-based vector safety, was the absence of serum-conversion of the sentinel animal and meaning that BoHV-4-A-gD_cp_gD_106_ΔTK or BoHV-4-CMV-IgK-gE2gD-TM did not spread between animals. This finding represents an important prerequisite for the use of this virus as a vaccine vector, in fact BoHV-4-A-gD_cp_gD_106_ΔTK and BoHV-4-CMV-IgK-gE2gD-TM could not be recovered from the blood and body secretes fluids of the inoculated animals. Therefore, it can be supposed that BoHV-4 replication take place only at the site of inoculation, at least in the goat, and BoHV-4 based vector is behaving as an un-competent replicating vector *in vivo* but competent replicating vector *in vitro*. Because we could not detect BoHV-4-base vector viremia in vaccinated animal, unlikely the vector is going to be present into the milk of lactating animals. However although unlikely, the fact that BoHV-4-base vector could be taken up by macrophages at the site of inoculum and delivered through the lymphatic rout to the mammary lymph-nodes, as well as to many other organs, could not completely excluded. The interesting data is that only one vaccinated animal developed a transient vaginal hyperemia on the 2^nd^ and 3^rd^ dpc consistently with the peak of virus titers shed (10^4^ – 10^2^TCID_50_/50 µl). Conversely, as expected, control goat developed typical vaginal lesions and severe signs which lasted longer than the viral shedding period. Vaccinated animals exhibited significantly different clinical scores from those of the control (P<0.01). After challenge vaccinated animals shed virus at reduced rate and for a much shorter period than the control goat. The mean AUC values for vaccinated and control goats were 9.08 and 38.12 (*P* = 0.015) respectively.

Several experimental vaccines were evaluated for their efficacy against CpHV-1 infection. All of them consisted of beta propiolactone inactivated CpHV-1 adjuvanted with Montanide [36] or MF59™ [25] and administered subcutaneously or given intravaginally (virus alone) [37] or adjuvanted with LTK63 mutant [38]. All of them were able to significantly protect the treated animals from the outcome of clinical signs. Protection from the infection was variable depending to the vaccine used. Even considering that the experiments have not been carried out concomitantly and animals could be of different ages and breed, the comparison of the AUC values of clinical scores recorded in the different experiments shows that of the efficacy of BoHV-4 based vector expressing CpHV-1 gD vaccine was comparable with that the inactivated MF59™ adjuvanted vaccine administered subcutaneously which totally clinically protected vaccinated animals from challenge virus (AUC values of 0.66 and 0, respectively). In addition, AUC values of clinical scores of animals intravaginally vaccinated with inactivated Montanide adjuvanted CpHV-1 (median AUC values 16) or with inactivated LTK3 adjuvanted CpHV-1 (median AUC values 9.7) were much higher than those reported in this study. MF59™ adjuvanted vaccine completely prevented virus shedding from vaccinated goats (AUC = 0). In the present experiment the vectored vaccine resulted in a higher reduction of virus shedding (AUC median value 9,08) than the inactivated vaccines administered mucosally (AUC median values 29,2 and 42,75 for vaccines given adjuvanted with LTK63 mutant or without adjuvant, respectively).

The data we obtained in the present work suggest that BoHV-4-A-gD_cp_gD_106_ΔTK expressing CpHV-1 gD alone can protect against CpHV-1 induced genital pathology and could potentially be used as a DIVA (Differentiating Infected from Vaccinated Animal) if combined with a CpHV-1 gE-specific ELISA, in light of the fact that BoHV-4 does not has a gE. Finally, the promising results and the proof-of-concept for the use of BoHV-4-based vector for goat vaccination presented in this study will pave the way to the production and testing of additional recombinant BoHV-4 derivatives as prototype vaccines targeting other important animal, and maybe, human pathogens.

## Materials and Methods

### Cells

Madin Darby Bovine Kidney [(MDBK) ATCC, CCL-22], bovine embryo kidney [(BEK) from Dr. M. Ferrari, Istituto Zooprofilattico Sperimentale, Brescia, Italy; (BS CL-94)], BEK expressing cre recombinase (BEK*cre*) [20] and Human Embryo Kidney 293T [(HEK 293T) ATCC, CRL-11268] cell lines were cultured with complete growth medium [Dulbecco's modified essential medium (DMEM) (SIGMA) containing 10% fetal bovine serum (FBS), 2 mM of l-glutamine, 100 IU/ml of penicillin (SIGMA), 100 µg/ml of streptomycin (SIGMA) and 2.5 µg/ml of Amphotericin B] and incubated at 37°C, 5% CO2 in a humidified incubator.

### Viruses

BoHV-4-A, BAC-BoHV-4-A, BAC-BoHV-4-A-gD_cp_gD_106_ΔTK, BoHV-4-A-gD_cp_gD_106_ΔTK, BAC-BoHV-4-A-CMV-IgK-gE2gD-TM, BoHV-4-CMV-IgK-gE2gD-TM, BoHV-4-EFGPΔTK, BoHV-4-A-LucΔTK, BoHV-1 (strain Oregon), and BVDV (strain NADL) were propagated by infecting confluent monolayers of BEK, BEKcre or MDBK cells at a multiplicity of infection (MOI) of 0.5 50% tissue culture infectious doses (TCID_50_) per cell and maintained in minimal essential medium (MEM; Sigma) with 2% FBS for 2 h. The medium was then removed and replaced with fresh MEM containing 10% FBS. When approximately 90% of the cell monolayer exhibited cytopathic effect (CPE) (72 h postinfection), the virus was prepared by freezing and thawing cells three times and pelleting the virions through 30% sucrose, as described previously [39]. Virus pellets were resuspended in cold MEM without FBS. TCID_50_ were determined with MDBK cells by limiting dilution.

The CpHV-1 strain BA.1 [40] was used for vaginal challenge and for the seroneutralization assay. The virus was cultivated in MDBK cells grown in DMEM. The viral titer was 10^6.5^TCID_50_/50 µl. For the challenge, the CpHV-1 stock was diluted to contain 10^5^TCID_50_/50 µl.

### Plasmids

The CpHV-1 gD 1249 bp ORF was amplified by PCR with a pair of primers primers (CpHV-1-sense, 5′cccccgctagcccaccatgtgggccctcgtgctcgcagcgctaagc3′; CpHV-1-antisense, 5′ccccgagtcgactcggggcagcgcgctgtagccgacgccgcc3′) introducing an NheI site (in the sense primer) and a PstI site (in the antisense primer) and subcloned in pJET vector (Fermentas) to generate pJET/gD_CpHV-1_. Next, CpHV-1 ORF fragment was cut out with NheI/SalI, inserted into pIgKE2VP2^2800^gD^106^ [26] depleted of IgKE2VP2^2800^ ORF by restriction digestion with NheI/SalI to generate pCMV-gD_cp_gD_106_. To generate pINT2-CMV-gD_cp_gD_106,_ pCMV-gD_cp_gD_106_ was cut with BamHI, blunted with T4, cut with NheI and the CMV-gD_cp_gD_106_ fragment was ligated in pINT2GFP [17] depleted of GFP by restriction digestion with NheI/SmaI.

### Transient transfection assay

Confluent Human Embryo Kidney 293T [(HEK 293T) ATCC, CRL-11268] cells in 6-well plates were transfected with pCMV-gD_cp_gD_106_ or pIgKE2VP2^2800^gD^106^, using LTX transfection reagent (Invitrogen) as suggested by the manufacturer. The transfection mixture was prepared in DMEM/high glucose without serum and antibiotics and left on the cells for 6 h at 37°C, 5% CO_2_, in a humidified incubator. After 6 h, the transfection mixture was replaced with complete medium (EMEM, 10% FBS, 50 IU/ml penicillin, 50 µg/ml streptomycin and 2.5 µg/ml Amphotericin B) and left to recover for 18 h at 37°C, 5% CO_2_ in air, in a humidified incubator. At 24 h post-transfection, cells were extracted to be analyzed by western immunoblotting.

### Western immunoblotting

Cell extracts containing 50 µg of total protein were electrophoresed through 10% sodium dodecyl sulfate-polyacrylamide gels and transferred to nylon membranes by electroblotting. Membranes were incubated with monoclonal anti-BoHV-1 gD (clone 1B8-F11; VRMD, Inc., Pullman, WA), probed with horseradish peroxidase-labeled anti-mouse immunoglobulin antibody (Sigma), and visualized by enhanced chemiluminescence (ECL kit; Pierce).

### Recombineering and selection

Recombineering was performed as previously described [20] with some modifications. Five hundreds microliters of a 32°C overnight culture of SW102 containing BAC-BoHV-4-A-KanaGalKΔTK [20], were diluted in 25 ml Luria–Bertani (LB) medium with or without chloramphenicol (SIGMA) selection (12.5 µg/ml) in a 50 ml baffled conical flask and grown at 32°C in a shaking water bath to an OD_600_ of 0.6. Then, 10 ml were transferred to another baffled 50 ml conical flask and heat-shocked at 42°C for exactly 15 min in a shaking water bath. The remaining culture was left at 32°C as the uninduced control. After 15 min the two samples, induced and uninduced, were briefly cooled in ice/water bath slurry and then transferred to two 15 ml Falcon tubes and pelleted using 5000 r.p.m. (eppendorf centrifuge) at 0°C for 5 min. The supernatant was poured off and the pellet was resuspended in 1 ml ice-cold ddH_2_O by gently swirling the tubes in ice/water bath slurry. Subsequently, 9 ml ice-cold ddH_2_O was added and the samples pelleted again. This step was repeated once more, the supernatant was removed and the pellet (50 µl each) was kept on ice until electroporated with gel-purified (3.9 kb fragment (TK-CMV-gDcpgD106-TK) obtained by cutting pINT2-CMV-gDcpgD106 with ClaI/PvuII (Fermentas). An aliquot of 25((l was used for each electroporation in a 0.1(cm cuvette at 25((F, 2.5(kV and 201 Ω. After electroporation, the bacteria were recovered in 1(ml LB (15(ml Falcon tube) for 1(h in a 32(C shaking water bath. For the counter selection step, the bacteria were transferred in 10(ml LB in a 50(ml baffled conical flask and incubated for 4.5(h in a 32(C shaking water bath. Bacteria serial dilutions were plated on M63 minimal medium plates containing 15(g/l agar, 0.2% glycerol (SIGMA), 1(mg/l d-biotin, 45(mg/l l-leucine, 0.2% 2-deoxy-galactose (DOG, SIGMA) and 12.5((g/ml chloramphenicol. Plates were incubated 3–5 days at 32(C. Several selected colonies were picked up, streaked on McConkey agar indicator plates (DIFCO, BD Biosciences) containing 12.5((g/ml of chloramphenicol and incubated at 32(C for 3 days until white colonies appeared. White colonies were grown in duplicate for 5–8(h in 1(ml of LB containing 50((g/ml of kanamycin or LB containing 12.5((g/ml of chloramphenicol. Only those colonies growing on chloramphenicol and not on kanamycin were kept and grown overnight in 5(ml of LB containing 12.5((g/ml of chloramphenicol. BAC DNA was purified and analyzed through HindIII restriction enzyme digestion for TK-CMV-gDcpgD106-TK fragment targeted integration. Original detailed protocols for recombineering can also be found at the recombineering website (http://recombineering.ncifcrf.gov).

### Cell culture electroporation and recombinant virus reconstitution

BEK or BEKcre cells were maintained as a monolayer with growth medium containing 90% DMEM, 10% FBS, 2 mM l-glutamine, 100 IU/ml penicillin and 10 µg/ml streptomycin. Cells were sub-cultured to a fresh culture vessel when growth reached 70–90% confluence (i.e., every 3–5 days) and were incubated at 37°C in a humidified atmosphere of 95% air–5% CO_2_. Plasmid DNA (5 µg) in 500 µl DMEM without serum was electroporated (Equibio apparatus, 270 V, 960 µF, 4-mm gap cuvettes) into BEK cells from a confluent 25-cm^2^ flask. Electroporated cells were returned to the flask, fed the next day, and split 1∶2 when they reached confluence at 2 days post-electroporation. Cells were left to grow until CPE appeared. Recombinant viruses were propagated by infecting confluent monolayers of MDBK cells at a m.o.i. of 0.5 TCID50 per cell and maintaining them in MEM with 10% FBS for 2 h.

### Goat skin primary culture

The primary goat skin cells were prepared from goat tail skin explants (goat: 1 years-old female) as previously described [41]. The explants were chopped into 2–3-mm^2^ pieces using sterile disposable scalpel and forceps. The pieces were minced on the surface of the 60-mm dishes (Falcon, B. D. Biosciences, Oxnard, CA, USA) and allowed to dry within the culture hood for 5–10 min to facilitate adherence of the pieces to the surface. Three milliliters of complete growth medium, which was maintained at 37°C prior to use, was then gently added from the side of the dish and the dishes were incubated at 37°C, 5% CO2 in a humidified incubator. When mixed populations of outgrowth cells reached confluence, to obtain separate stromal and epithelial cell populations, the cell were detached from the wells by standard trypsin treatment, plated at a density of 1×10^5^ cells in 2 ml per well using 24-well plates (Nunc) and then removed 3 hs after plating, which allowed selective attachment of stromal cells. The removed cell suspension was then replated and incubated allowing epithelial cells to adhere. Stromal and epithelial cell populations were distinguished by cell morphology. Cells were infected with BoHV-4EGFPΔTK or BoHV-4-A-gD_cp_gD_106_ΔTK at 1 M.O.I..

### Organotypic culture preparation and infection

Goat skin organotypic cultures were prepared with similar procedures used for primary cell cultures preparation. Goat tail skin explants (goat: 1 years-old female) were chopped into 5–10-mm^2^ pieces using sterile disposable scalpel and forceps. The pieces were minced on the surface of the 60-mm dishes (Falcon, B. D. Biosciences, Oxnard, CA, USA) and allowed to dry within the culture hood for 5–10 min to facilitate adherence of the pieces to the surface. Three milliliters of complete growth medium, which was maintained at 37°C prior to use, was then gently added from the side of the dish and the dishes were incubated at 37°C, 5% CO2 in a humidified incubator. Organotypic cultures were infected with 10^5^ TCID50 of BoHV-4-A-LucΔTK.

### In vivo Bioluminescence Imaging (BLI)

*In vivo* imaging was performed using an IVIS imaging system (Caliper Life Sciences, Alameda, CA). After 48 hours of subcutaneous BoHV-4-A-LucΔTK injection the injected area was surgically dissected following local anaesthesia and dipped in luciferin solution (Promega) for 15 minutes. Photons emitted were quantified using Living Image® software (Caliper Life Sciences, Alameda, CA).

### Viral growth curves and plaque assay

BEK cells were infected with BoHV-4-A and BoHV-4-A-gD_cp_gD_106_ΔTK at an M.O.I. of 1 TCID_50_/cell and incubated at 37°C for 4 h. Infected cells were washed with serum-free EMEM and then overlaid with EMEM containing 10% FBS, 2 mM l-glutamine, 100 IU/ml penicillin (Sigma), 100 µg/ml streptomycin (Sigma) and 2.5-µg/ml Amphotericin B. The supernatants of infected cultures were harvested after 12, 24, 36, 48, 60, 72, 96 and 120 h, and the amount of infectious virus was determined by limiting dilution on BEK cells.

### Serum-neutralization test

Serum neutralization tests were performed as follows. Twenty-five microliters of each serum sample were added to the first line of wells of 96-well plates. Twenty-five microliters of DMEM were added to each well and, for each serum tested, serial twofold dilutions were made. Positive and negative serum controls were included. Twenty-five microliters of virus suspension containing 100 TCID50 of CpHV-1, BVDV or BoHV-1 were added to each well. After 1 h of incubation at 37°C, 50 µl of a MDBK cell suspension were added to each well and the plates were incubated for 3 days at 37°C in a humidified atmosphere of 95% air–5% CO_2_. Expression of viral infectivity and serum neutralizing activity through CPE were detected by microscopy and or by crystal violet staining of the cell monolayer. The neutralization antibody titer was expressed as the reciprocal (log 2) of the final dilution of serum that completely inhibited viral infectivity.

### Animal study

For preliminary immunization study, seven adult female crossbred goats were employed. For the challenge study, four adult female crossbred goats were employed. Before immunization the goats were tested to insure that they were negative for serum VN antibodies against BoHV-1, BVDV and CpHV-1.

The experiments were approved and authorized by the Ethical Committee of the University of Bari and the Italian Ministry of Health and was carried out at the University of Bari according to the National Guide for Care and Use of Experimental Animals (n. 4/2012).

The animals were kept under observation for 20 days and examined daily for general and local clinical signs (including body temperature, vaginal hyperemia, oedema, vesicular lesions and pain). A cumulative clinical score was determined in each goat by grading the clinical signs as follows: 0, absent; 1, mild; 2, moderate; 3, severe. Temperature increments above normal were graded as follows: >0.5–1°C = 1; >1.1–1.5°C = 2; >1.5°C = 3 [42].

In order to evaluate the viral shedding, vaginal swabs were daily collected from all the goats. The swabs were placed in 1.5 ml of DMEM, centrifuged at 10,000 rpm for 5 min and then the supernatant was collected, treated with 0,1 ml of an antibiotic mixture (5,000 IU/penicillin, 2,500 µg/ml streptomycin and 10 µg/ml amphotericin B) and incubated for 30 min at room temperature. Each sample was serially diluted (10-fold) and inoculated in quadruplicate onto MDBK cells placed in 96-well microtiter plates (Corning). Plates were incubated for 3 days at 37°C with 5% CO_2_ and then the virus titer was measured as previously described [43].

A Real-Time PCR assay was used to quantitate CpHV-1 genomic DNA in vaginal swabs. The assay was performed as previously reported [44]. Blood samples were drawn at T0, T7, T15, T25, T32 and processed for seroneutralization test as previously reported [36].

### Data Analysis

The data were analyzed by R software (version 2.8.1) by calculating the area under the curve (AUC). All hypothesis test were conducted at the 0.05 level of significance (two-sides). Clinical score data and vaginal virus titers shed from goats was calculated for each animal and each group (vaccinates and control) and the statistical significance was evaluated using Mann-Whitney test.
